# Devising novel performance measures for assessing the behavior of multilayer perceptrons trained on regression tasks

**DOI:** 10.1371/journal.pone.0285471

**Published:** 2023-05-18

**Authors:** Giuliano Armano, Andrea Manconi

**Affiliations:** 1 Dept of Mathematics and Computer Science, University of Cagliari, Cagliari, Italy; 2 Institute of Biomedical Technologies - National Research Council, Segrate, MI, Italy; California State University, Long Beach, UNITED STATES

## Abstract

This methodological article is mainly aimed at establishing a bridge between classification and regression tasks, in a frame shaped by performance evaluation. More specifically, a general procedure for calculating performance measures is proposed, which can be applied to both classification and regression models. To this end, a notable change in the policy used to evaluate the confusion matrix is made, with the goal of reporting information about regression performance therein. This policy, called generalized token sharing, allows to a) assess models trained on both classification and regression tasks, b) evaluate the importance of input features, and c) inspect the behavior of multilayer perceptrons by looking at their hidden layers. The occurrence of success and failure patterns at the hidden layers of multilayer perceptrons trained and tested on selected regression problems, together with the effectiveness of layer-wise training, is also discussed.

## 1 Introduction

Multilayer perceptrons (MLPs) are a technique of paramount importance for machine learning, also considering the renewed interest for artificial neural networks (ANNs) advocated by deep learning (on this matter see in particular the foundational article of Bengio [1]). Following the recommendations of explainable AI [2], highlighting the semantics that operates behind the scenes in various AI systems and techniques, including ANNs and deep networks, is also a goal of prominent interest. Several works framed along this perspective have been made in recent years. Without the claim of being exhaustive, let us recall some relevant proposals. In 2000 Tishby et al. [3] introduce the concept of Information Bottleneck (IB) –strictly related to a corresponding constrained optimization problem that generalizes the rate distortion theory. Among the publications issued on this matter, let us cite in particular [4], in which deep neural networks are analyzed via the IB theoretical framework, pointing to the mutual information that can be measured between the layers and the input and output variables. Subsequently, Shwartz and Tishby [5] revisit the concept of IB and demonstrate the effectiveness of the Information-Plane visualization for deep neural networks. The authors also point out that at any hidden layer an ANN can be divided in two parts, i.e., an encoder and a decoder. In particular, the encoder can be seen as a tool able to project the given input data onto an alternative feature space, according to the weights and to the activation functions that occur therein. Not disconnected from this conceptualization, some studies have been carried out on layer-wise training –which consists of training one layer at a time. Besides, this training strategy allows to overcome the vanishing gradient problem [6], as here error signal backpropagation affects only a single hidden layer at a time. After the advent of deep learning, several training strategies have been proposed that embed layer-wise training as preliminary step. For example, Bengio et al. [7] adopt a pre-training greedy layer-wise training strategy, Kulkarni and Karande [8] use kernel similarity, and Wulff et al. [9] use simultaneous perturbation stochastic approximation. In 2020 Armano [10] has shown that layer-wise training can be used in fact as stand-alone training strategy, pointing that its performance is typically at least as good as the one obtained with the classical backpropagation algorithm.

Going back to the central topic of explainable AI, in 2018 Armano [11] tries to disclose the inner semantics that regulates the training of an MLP by investigating the occurrence of relevant patterns, in particular success and failure, on hidden layers. To this end, 〈*φ*, *δ*〉 measures and diagrams [12] are used, for their ability to illustrate the characteristics of each hidden layer as it were in fact an alternative input source for the subsequent layers. According to this view, the rules for evaluating the importance of input features, defined for binary classification problems with binary or real-valued features could also be used for evaluating the importance of neurons that occur at the hidden layers of an MLP [11, 12]. In its standard formulation, the 〈*φ*, *δ*〉 measure of feature importance, advocated by a policy called *token sharing* (say TS hereinafter), could not be applied to regression problems –due to the apparent unfeasibility of calculating a confusion matrix (*CM* hereinafter) for this kind of tasks.

Fortunately, classification and regression tasks share some common characteristics, which enables to frame them in a common scheme. The main focus of this article is on how to calculate the *CM* for regression problems. To this end, the TS policy is generalized, thus allowing on these models a) to assess the overall performance, b) to evaluate the importance of input features, and c) to inspect the behavior of MLPs by looking at their hidden layers. The occurrence of success or failure patterns found at the hidden layers of MLPs trained on regression problems is also investigated. The remainder of this article is organized as follows: after preliminary definitions, Section 2 is focused on the adopted materials and methods. In particular, it reports some basic concepts regarding the analysis of input and hidden layers of an MLP, performed by means of 〈*φ*, *δ*〉 measures and diagrams. Special care is also devoted to show how TS can be generalized to allow its application to regression problems. After pointing to the characteristics of the experimental benchmark, which include some details on the layer-wise training strategy (i.e., progressive training) adopted to foster the analysis of MLP hidden layers, Section 3 analyzes some experimental results. Section 4 discusses relevant aspects and issues regarding the proposal. Finally, Section 5 draws conclusions and sketches future work.

## 2 Materials and methods

First, this session discusses the main contribution and motivations of the proposed approach. Subsequently, after having provided some preliminary definitions aimed at facilitating the understanding of the underlying concepts and solutions, the attention moves to more technical details, concerning in particular the ability to evaluate the internal behavior of MLPs trained on regression problems.

### 2.1 Main contribution and motivations of the proposed approach

As pointed out, the main focus of this article is on how to calculate the *CM* for regression problems. On the one hand, this aim appears more as a contradiction in terms than as an achievable goal, to the point that at a first glance it may look like a misplaced question. In fact, this perspective is definitely far from a typical binary classification setting to which the calculus of *CM* applies. On the other hand, TS could be considered a first step towards solving the problem, due to its ability to handle real-valued features. To better explain this concept, let us focus on the inherent characteristics of the involved quantities, with varying the perspective.

In binary classification problems, both features and target are binary (with values typically encoded as false/true, 0/1 or −1/+1). This is the perfect setting for calculating the *CM*, as the policy for increasing true/false positives/negatives in this case is straightforward. A problem characterized by real-valued features and binary target stands half-way between a binary classification and a regression setting, as here the target is still binary whereas features are real-valued. An effective policy (i.e., TS) aimed at updating the *CM* has been devised and implemented to take action also on this kind of problems. Hence, there is apparent room for adapting TS to the more general setting even when the target is real-valued.

This methodological article is mainly dedicated to illustrate that this generalization attempt is viable. Thanks to the proposed generalization of the TS policy, called *generalized token sharing* (GTS hereinafter), the most important performance evaluation steps can be easily carried out also on regression tasks. Indeed, the result of this effort has gone beyond any wildest expectation, as the proposed measure, devised to deal with regression problems, includes as special cases both TS and the classical method for calculating *CM* on binary classification problems.

### 2.2 Common groundwork on 〈*φ*, *δ*〉 measures and diagrams

Table 1 establishes a common groundwork for the symbols used throughout this article. Relevant definitions, all derived from the *CM*, are briefly reported therein. As for 〈*φ*, *δ*〉 measures, they are defined on top of specificity and sensitivity as follows:

$$\begin{array}{c} \left\{ \begin{array}{cc} \varphi & =\rho -\bar{\rho }\\ \delta & =\rho +\bar{\rho }-1 \end{array}\right. \end{array}$$ (1)

Notably, *φ* typically denotes an estimate of the bias of a classifier with respect to the negative or positive category, whereas *δ* represents the accuracy stretched in the interval [−1, +1]. Both the cited measures are in fact “unbiased”, meaning that the corresponding dataset is assumed to be perfectly balanced. Besides, showing that *δ* represents the unbiased accuracy stretched in the interval [−1, +1] is straightforward. In symbols (with *a*_*u*_ denoting the unbiased accuracy):

$$\begin{array}{c} \delta =\bar{\rho }+\rho -1=2\cdot \frac{\bar{\rho }+\rho }{2}-1=2\cdot \frac{tn+tp}{2}-1=2\cdot a_{u}-1 \end{array}$$ (2)

A 〈*φ*, *δ*〉 diagram is based on the above definitions, with *φ* as *x*-axis and *δ* as *y*-axis. Relevant properties of these diagrams are:

- the *φ*-axis is the locus of points for which entropy reaches its maximum (or, equivalently, mutual information reaches its minimum);
- the *δ*-axis is the locus of points for which specificity equals sensitivity (i.e., it is the locus of breakeven points);
- Due to the limits imposed on specificity and sensitivity, the 〈*φ*, *δ*〉 space is characterized by the equation |*φ*| + |*δ*| ≤ 1, which gives rise to a characteristic *diamond shape*.

**Table 1:** Summary of useful symbols used throughout the article.

Symbol		Definition	
*N*	=	number of negatives	
*P*	=	number of positives	
*TN*	=	number of true negatives	
*FP*	=	number of false positives	
*FN*	=	number of false negatives	
*TP*	=	number of true positives	
*n*	=	percent of negatives	= *N*/(*N* + *P*)
*p*	=	percent of positives	= *P*/(*N* + *P*)
*tn*	=	percent of true negatives	= *TN*/*N*
*fp*	=	percent of false positives	= *FP*/*N*
*fn*	=	percent of false negatives	= *FN*/*P*
*tp*	=	percent of true positives	= *TP*/*P*
$\bar{\rho }$	=	specificity	≡ *tn*
*ρ*	=	sensitivity	≡ *tp*
*a*	=	accuracy	= *n* ⋅ *tn* + *p* ⋅ *tp*
*a*_*u*_	=	unbiased accuracy	= *a*|_*n*=*p*=1/2_ = (*tn* + *tp*)/2

〈*φ*, *δ*〉 diagrams have been initially devised for assessing classifier performance and for computing the so-called class signature over the input features. In the former case, the *CM* for the classifier under testing is evaluated starting from the results obtained in one or more tests, and then the performance in terms of *φ* and *δ* is shown in a 〈*φ*, *δ*〉 diagram. In the latter case, the *CM* obtained by treating each feature as it were in fact an elementary (i.e., single-feature) classifier is evaluated, and then *all* the corresponding results are scattered in a 〈*φ*, *δ*〉 diagram.

**Fig 1:**
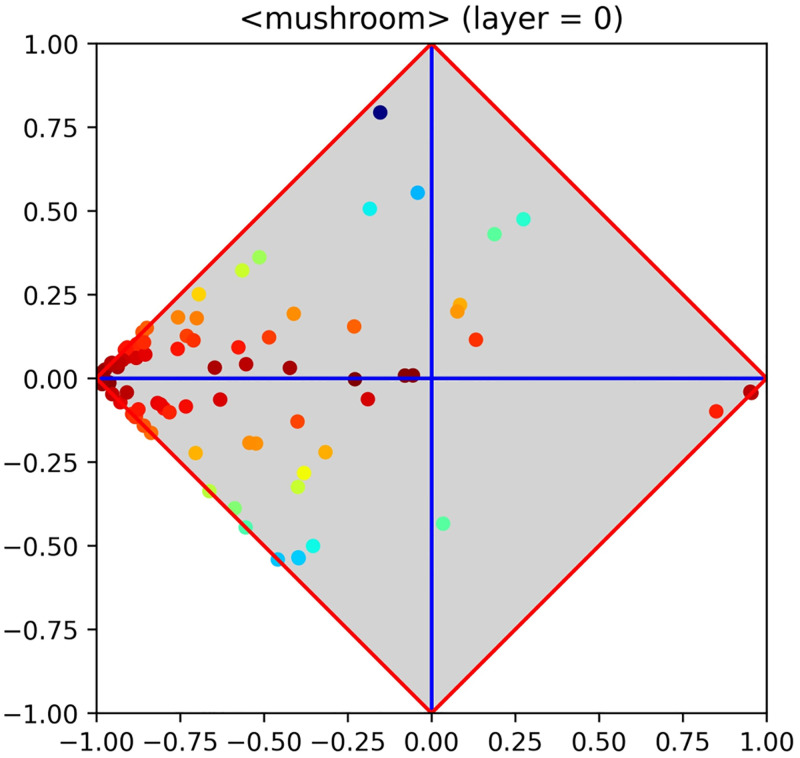
Class signature of the toy dataset *mushroom*, from UCI. The problem is expected to be easy, as several features are in medium or high agreement with the positive or negative category.

As an example, Fig 1 reports the class signature evaluated on the toy dataset *mushroom*, from the machine learning repository of the University of California at Irvine, (say UCI, hereinafter). This signature clearly highlights that the problem is expected to be easy, as several features are in medium or high agreement with the positive (i.e., edible) or negative (i.e., poisonous) category. Covariant features (i.e., those in agreement with the positive category) lie close to the upper corner, whereas contravariant ones (i.e., those in agreement with the negative category) lie close to the lower corner. In either case, the closer the better. Notably, both highly covariant and highly contravariant features are important, due to their highly discriminant capability (see also [11] for more information on this matter).

### 2.3 Introducing the GTS policy

In order to apply the 〈*φ*, *δ*〉 analysis to regression problems, a critical issue must be dealt with in advance –i.e., finding rules capable of allowing the construction of a *CM* also in presence of real-valued targets. To better understand the issue, let us summarize the relevant information that constrains and defines the problem. The starting point is that a 〈*φ*, *δ*〉 diagram is built upon 〈*φ*, *δ*〉 measures, which in turn are linear combinations of specificity and sensitivity. As a consequence, *φ* and *δ* can be calculated only if the *CM* is available. Fortunately, the classical concept of *CM* can be rethought and generalized to the point of making its evaluation possible even for regression problems. This subsection is devoted to illustrate this generalization process, first recalling the classical definition of *CM* (in which a unitary token is assigned to either *TN*, *FP*, *FN* or *TP*), then looking back at the concept of TS, and finally concentrating on how to deal with real-valued targets.

As pointed out, there are three core settings over which the concept of *CM* can be declined: a) binary target with binary features, b) binary target with real-valued features, c) real-valued target with real-valued features. Despite their apparent diversity, in fact all cases can be analyzed according to a unifying perspective. In particular, a proper policy must be devised to share a unitary token, for each example, among *TN*, *FP*, *FN* and *TP*. Due to the constraints that apply to each specific setting, it is widely acknowledged that in case (a) the token is used to update only *one* member of the *CM*. In case (b) the token is *shared* between either *TN* and *FP* or between *FN* and *TP*, depending on the target value. As for case (c), it will be shown that –in principle– the token may be shared among *all* *TN*, *FP*, *FN*, and *TP*. It is worth pointing out in advance that case (a) is generalized by case (b), which in turn is generalized by case (c). For the sake of clarity, all settings are analyzed separately, from the less to the most general one, but only after reporting some preliminary definitions that apply to all cases. These definitions are:

- *y* Variable denoting the target (with *t* generic value)
- *f* Variable denoting a feature (with *v* generic value)
- Δ_*t*,*v*_ Linear distance between *t* and *v*, namely |*t* − *v*|
- *m*_*t*,*v*_ Measure of similarity between *t* and *v*, namely 1 − Δ_*t*,*v*_

*Case (a): Token assignment for binary problems with binary features.* The simplest case occurs when target and feature values are binary. This setting describes the classical scenario, in which the *CM* is updated –ideally step by step– by identifying *which* one among *TN*, *FP*, *FN*, and *TP* should be increased. Without loss of generality, let us assume that features are scaled in the range [−1, +1], whereas the value −1 or +1 can be given to the target, with the canonical association of −1 ⇒ *False* and +1 ⇒ *True*.

Table 2 highlights all cases that may occur under this setting, reporting also the distance Δ_*t*,*v*_ and the measure of similarity *m*_*t*,*v*_. Note that, by definition, *m*_*t*,*v*_ can be +1 or −1, depending on whether the target and the feature at hand coincide or not. The table clearly highlights that the token is delivered to only one among *TN*, *FP*, *FN*, and *TP*. The selection of the side to which the token is assigned (i.e., negative or positive category) depends on the sign of the target. By definition, at the end of the computation, *TN* + *FP* = *N*, *FN* + *TP* = *P*, and *tn* + *fp* = *fn* + *tp* = 1.

**Table 2:** Policy adopted for updating the *CM* under the basic setting in which both target and features are binary. The side for *CM* update is given by the sign of the target.

*y*	*f*	Δ_*t*,*v*_	*m*_*t*,*v*_	Policy for *CM* update		Side
−1	−1	0	+1	*TN* += 1	*FP* += 0	negative
−1	+1	2	−1	*TN* += 0	*FP* += 1	
+1	−1	2	−1	*FN* += 1	*TP* += 0	positive
+1	+1	0	+1	*FN* += 0	*TP* += 1	

*Case (b): Token sharing for binary problems with real-valued features.* In this setting the binary problem is relaxed by allowing real-valued features, meaning that the target can be −1 or +1, whereas feature values are now in [−1, +1]. The equations reported in Table 3 illustrate how Table 2 can be generalized for dealing with real-valued features.

**Table 3:** Policy adopted for updating the *CM* under the setting in which the target is binary and features are real-valued. The side for *CM* update is given by the sign of the target.

*y*	*f*	Δ_*t*,*v*_	*m*_*t*,*v*_	Policy for *CM* update	Side
−1	*v*	1 + *v*	−*v*	*TN* += (1 − *v*)/2	negative
				*FP* += (1 + *v*)/2	
+1	*v*	1 − *v*	+*v*	*FN* += (1 − *v*)/2	positive
				*TP* += (1 + *v*)/2	

Note that, although with the same semantics seen for the binary case, Δ_*t*,*v*_ and *m*_*t*,*v*_ are now continuously varying between −1 and +1. Table 3 clearly highlights that the token is *shared* among either *TN* and *FP* or *FN* and *TP*. The selection of the side to which the token is delivered still depends on the sign of the target. It is easy to verify that the rules for *CM* update reported in Table 3 imply the ones reported in Table 2. In this case any single update of *TN* and *FP* sums up to 1, and the same holds for *FN* and *TP*, otherwise the frequentist interpretation of *CM* would fail. Hence, TS still guarantees that at the end of the computation *TN* + *FP* = *N* and *FN* + *TP* = *P*, which in turn implies that *tn* + *fp* = *fn* + *tp* = 1.

**Fig 2:**
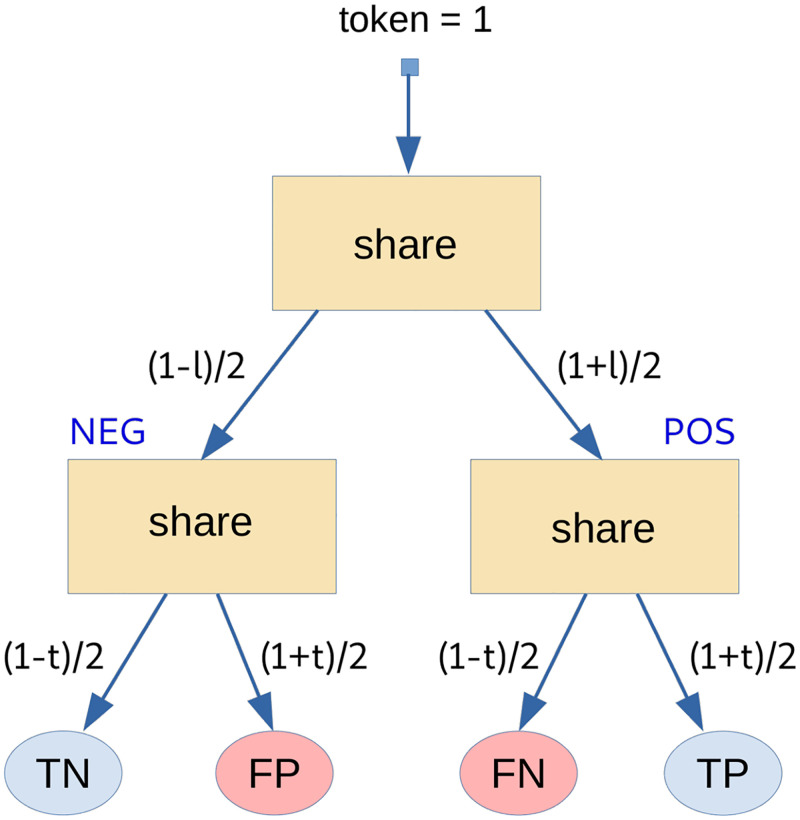
Graphical summary of the GTS process that occurs (in two steps) when target and features are real-valued. It is easy to verify that, by construction, the reported schema implies the previous TS schemas. In particular, a) *t* = ±1 and *v* = ±1 correspond to binary target with binary features; b) *t* = ±1 and *v* ∈ [−1, +1] correspond to binary target with real-valued features; whereas c) *t*, *v* ∈ [−1, +1] is for real-valued target and features.

*Case (c): Token sharing for problems characterized by real-valued target and features.* In this setting the problem is further relaxed by allowing also a real-valued target, so that both target and feature values now lie in the interval [−1, +1]. Fig 2 highlights that, in the most general case, token sharing is performed in two steps.

The first step consists of splitting the unitary token into a negative and positive part (say *R*_⊖_ and *R*_⊕_) according to the current target value. In symbols:

$$\begin{array}{c} \left\{ \begin{array}{cc} R_{⊖} & =\;\;(1-t)/2\\ R_{⊕} & =\;\;(1+t)/2 \end{array}\right. \end{array}$$ (3)

Note that, as expected, *t* = +1 implies *R*_⊖_ = 0 and *R*_⊕_ = 1, whereas *t* = −1 implies *R*_⊖_ = 1 and *R*_⊕_ = 0. The second step is entrusted with delivering *R*_⊖_ and *R*_⊕_ according to the distance between *t* and *v*. The corresponding formulas are reported in Table 4, which highlights that in principle the update may affect *all* components of the *CM*.

**Table 4:** Policy adopted for updating the *CM* under the relaxed setting in which target and features are real-valued. The policy for *CM* update makes clear that both sides of the matrix can be affected by a change. Note also that the general definitions of Δ_*t*,*v*_ and *m*_*t*,*v*_ are reported here, due to the absence of constraints on *t* and *v*.

*y*	*f*	Δ_*t*,*v*_	*m*_*t*,*v*_	Policy for *CM* update	Side
*t*	*v*	|*t* − *v*|	1—Δ_*t*,*v*_	$TN+=R_{⊖}\cdot (1-\frac{\Delta_{t,v}}{2})$	negative
				$FP+=R_{⊖}\cdot \frac{\Delta_{t,v}}{2}$	
*t*	*v*	|*t* − *v*|	1—Δ_*t*,*v*_	$FN+=R_{⊕}\cdot \frac{\Delta_{t,v}}{2}$	positive
				$TP+=R_{⊕}\cdot (1-\frac{\Delta_{t,v}}{2})$	

It is easy to verify that the rules for *CM* update reported in Table 4 imply the ones reported in Table 3 and hence those reported in Table 2. Moreover, also GTS guarantees, by construction, that at the end of the computation *TN* + *FP* = *N* and *FN* + *TP* = *P* and that *tn* + *fp* = *fn* + *tp* = 1.

## 3 Results

This section reports some experimental results focused on how to use 〈*φ*, *δ*〉 diagrams to assess MLP models trained on regression tasks. The rise of success and failure patterns inside MLPs is also briefly discussed. Experiments have been run using MLPs equipped with more than one hidden layer and trained on relevant datasets downloaded from UCI. The adopted training algorithm is progressive training [10], which implements a layer-wise strategy that is immune, by definition, from the vanishing (or exploding) gradient problem. To facilitate the reader, first a few comments on progressive training are given, followed by some recommendations on how to use 〈*φ*, *δ*〉 diagrams to assess MLP models trained on regression tasks. Experimental results on the selected datasets are discussed afterwards.

### 3.1 Brief summary on progressive training

As pointed out, nothing prevents from thinking of an MLP as a device that at each hidden layer projects the input onto an alternative space, whose dimensions coincide with the number of neurons that occur therein. Hence, any training algorithm is expected to perform an overall transformation aimed at “adapting” the input features to the desired output. In principle, this process is different for classification and regression. However, neural architectures often treat both kinds of problems in the same way, meaning that the information used to update weights is typically the difference between the expected output and the actual one, regardless of the kind of problem at hand.

Progressive training has been devised according to this view, and following the insight that an MLP can always be seen as made up of two parts: an encoder and a decoder. For example, let us assume that an MLP equipped with three hidden layers –say *H*_1_, *H*_2_, and *H*_3_– must be trained on a given dataset. With *L*_*in*_ and *L*_*out*_ input and output layer, respectively, one can assert that the pipeline 〈*L*_*in*_, *H*_1_, *H*_2_, *H*_3_〉 provides an alternative representation of the input for the last layer of the MLP. Hence, in this case everything goes as if there is a “line” drawn between *H*_3_ and *L*_*out*_ such that all layers on the left act as encoder, whereas the output layer as decoder.

Most of the algorithms based on backpropagation consider an MLP as a whole, meaning that all layers are trained concurrently. On the contrary progressive training provides a layer-wise training strategy, driven by the desired output. Following this insight, the training starts off with a gregarious MLP consisting of 〈*L*_*in*_, *H*_1_, *L*_*out*_〉. Then *H*_1_ is “frozen”, meaning that it cannot be further modified, and another gregarious MLP consisting of 〈*L*_*in*_, *H*_1_, *H*_2_, *L*_*out*_〉 is trained. The same procedure is put into practice on *H*_3_, thus ending the training process in this case. More generally, the training process ends when all hidden layers of the MLP have been trained.

### 3.2 Assessing MLP models by means of 〈*φ*, *δ*〉 diagrams

The GTS policy has made feasible to investigate the inner behavior of MLPs trained on regression problems. Depending on the change of perspective enforced by GTS, the term *model signature* will be used hereinafter instead of class signature (let us recall that the class signature consists of calculating a 〈*φ*, *δ*〉 pair for each feature of the problem at hand and then scattering the result on a corresponding diagram). As done for classification problems, the model signature can be evaluated also on the hidden layers of an MLP, thus getting information about the effectiveness of the training process. As for the output layer, the 〈*φ*, *δ*〉 placement of the corresponding neuron highlights the overall performance of the MLP at hand; in particular, its *δ* component shows the performance of the prediction, the closer to the upper corner the better.

**Fig 3:**
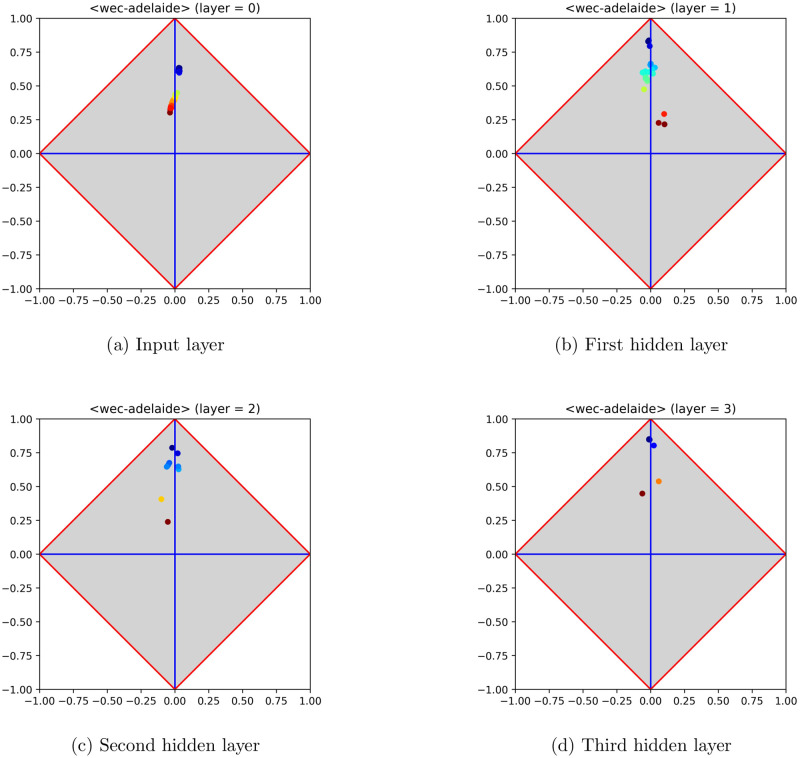
Model signature regarding the input layer and the hidden layers of an MLP trained on the dataset *Wave Energy Converters*, from UCI. Signatures are reported from left to right and from top to bottom (the input signature lies at the top left of the figure). The sequence of signatures clearly highlights that MLP layers are able to approximate quite well the given signal. This ability is made clear from the first hidden layer on, meaning that the problem is easy and that further layers are in fact redundant.

The results illustrated and discussed in this section confirm the validity of the 〈*φ*, *δ*〉 analysis, pointing out that relevant patterns can be found also on MLPs trained on regression problems. Let us consider, for instance, the dataset *Wave Energy Converters* [13], taken from UCI, which contains positions and absorbed power outputs of wave energy converters (WECs) in four real-world scenarios from the southern coast of Australia (i.e., Sydney, Adelaide, Perth and Tasmania). Fig 3 reports the model signature regarding the input layer and the hidden layers of an MLP trained on this dataset (for the sake of simplicity, only the part regarding Adelaide has been used to perform experiments).

**Fig 4:**
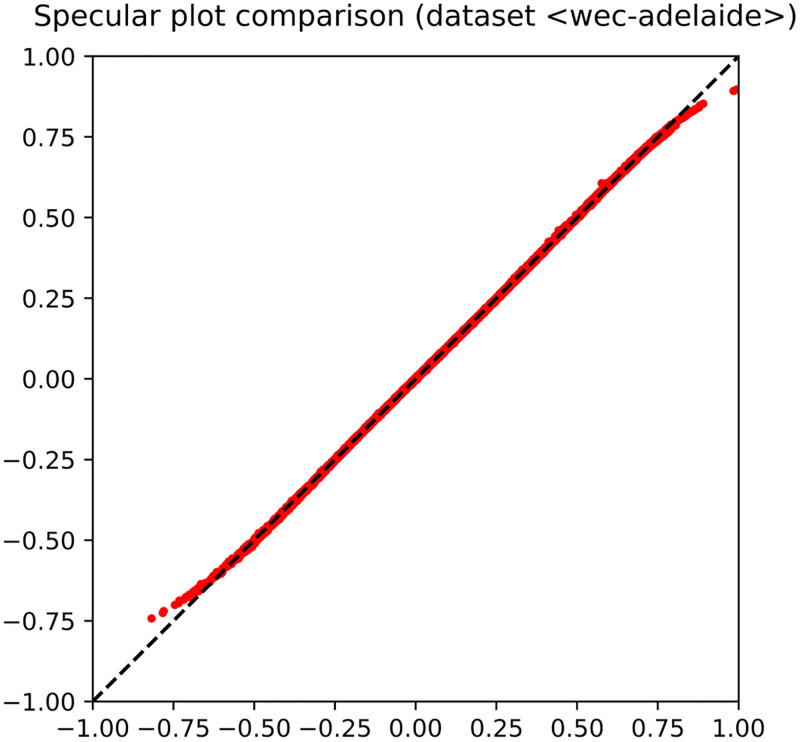
Observed-vs-predicted diagram that illustrates the fitting obtained by the MLP on the dataset *Wave Energy Converters*.

The shape of the trained MLP consists of 〈48, 20, 10, 5, 1〉 neurons, 48 being the number of input features. The input signature (top left of the figure) highlights that the problem is in fact easy to solve, as several neurons lie close to the upper corner. Let us recall that a feature lying exactly at the upper corner would mean that it is coincident with the target. As for the hidden layers, it is clear that the MLP has been able to attain a good fitting with the target, since several neurons occur close to the upper corner. This pattern of generalization success is already occurring at the first hidden layer, so that the subsequent hidden layers could even be removed, being not expected to improve the overall performance. The performance has also been reported in an observed-vs-predicted diagram, in which targets lie on the *x*-axis and predicted output on the *y*-axis. The diagram shown in Fig 4 highlights that MLP output and target are almost coincident, as pointed out by their occurrence very close to the straight line at 45 degrees that characterizes a perfect fitting.

### 3.3 Experimental results on relevant datasets

Beyond the identification of relevant patterns, some experimental results are reported in this section. The list of datasets used for experiments (downloaded from UCI) follows hereinafter:

- Abalone (Tasmania abalone age prediction);
- Airfoil (NASA Airfoil self-noise);
- Boston housing (Boston housing data from StatLib library);
- CBM (Condition Based Maintenance of data propulsion plants);
- EGrid stability (Electrical grid stability simulated data);
- Traffic (Behavior of the urban traffic of the city of Sao Paulo in Brazil);
- Bike sharing (Daily bike sharing at Porto, Portugal);
- WEC (Wave Energy Converters on southern coast of Australia).

The experimental benchmark has been set as follows: 10 train and test runs have been performed on each dataset. At each run, 70% of available examples have been selected for training, using random sampling without replacement; then performance has been assessed on the remaining 30% focusing on *R*2 and *δ*. Table 5 reports average results for each dataset, including some basic information, i.e., number of instances, number of features and the output signal selected for regression (i.e., the target).

**Table 5:** Performance on regression tasks as shown by MLPs trained on some exemplar UCI datasets. After reporting relevant information for each dataset (i.e., number of instances, number of features and the output signal selected for regression), the table shows the performance measured in terms of *R*2 and *δ*. The difference (in absolute value) between *R*2 and *δ* has also been reported. Experiments have been performed by repeating 10 training and test runs (each with 70% and 30% of available examples), followed by averaging. Each test has been performed with random sampling without replacement. Problems are also labeled with one or two white circles (standing for low and medium difficulty, respectively).

	Dataset Info			Performance			
Dataset	Instances	Feat.	Target	*R*2	*δ*	|*R*2 − *δ*|	
Abalone	4,177	8	shell rings	0.56	0.32	0.24	∘∘
Airfoil	1,503	6	sound level	0.68	0.71	0.03	∘∘
Boston hous.	506	13	house price	0.77	0.85	0.08	∘∘
CBM	11,934	17	ship speed	1.00	1.00	0.00	∘
EGrid stabil.	10,000	12	stability	0.42	0.47	0.05	∘∘
Traffic	135	17	slowness	0.73	0.40	0.33	∘∘
Bike sharing	731	15	total count	0.99	0.98	0.01	∘
WEC	71,999	48	total power	1.00	1.00	0.00	∘

Depending on the performance in terms of fitting, each dataset has been labeled with one or two white circles (standing for low and medium difficulty, respectively). It is worth noting that *R*2 and *δ* show full agreement on easy problems, whereas they differ for problems of medium difficulty. This should not be surprising, as *R*2 is quadratic and *δ* is linear. In fact, the former is based on the residual sum of squares, normalized by the variance of data, whereas the latter comes from a linear combination of specificity and sensitivity. However, despite these differences, they are both able to highlight the difficulty or easiness of a problem.

As pointed out, 〈*φ*, *δ*〉 diagrams can also be used to provide information about the ability of an MLP to fit the training data. An analysis performed on the hidden layers of the MLPs used for experiments has shown –on average– the occurrence of relevant patterns. In particular, two clusters of datasets could be clearly identified. The first (consisting of *Boston housing*, *Bike sharing*, *WEC*, and *CBM*) is characterized by high performance. As common characteristic, the hidden layers of an MLP trained on any of these datasets show the occurrence of success patterns similar to the ones reported for the *WEC* dataset. Conversely, the remaining datasets are characterized by lower performance and absence of success patterns.

**Fig 5:**
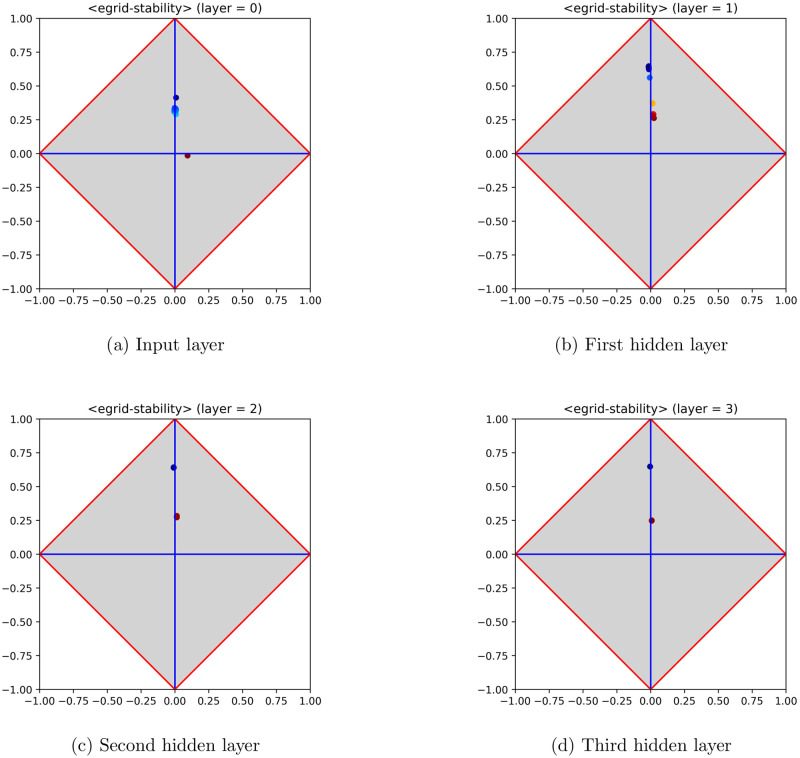
Model signature regarding the input layer and the hidden layers of an MLP trained on the dataset *EGrid stability*, from UCI. Signatures are reported from left to right and from top to bottom (the input signature stands at the top left of the figure). The sequence of diagrams makes clear the inability of the MLP to come up with a good approximation of the actual function, as negligible improvements occur across hidden layers.

**Fig 6:**
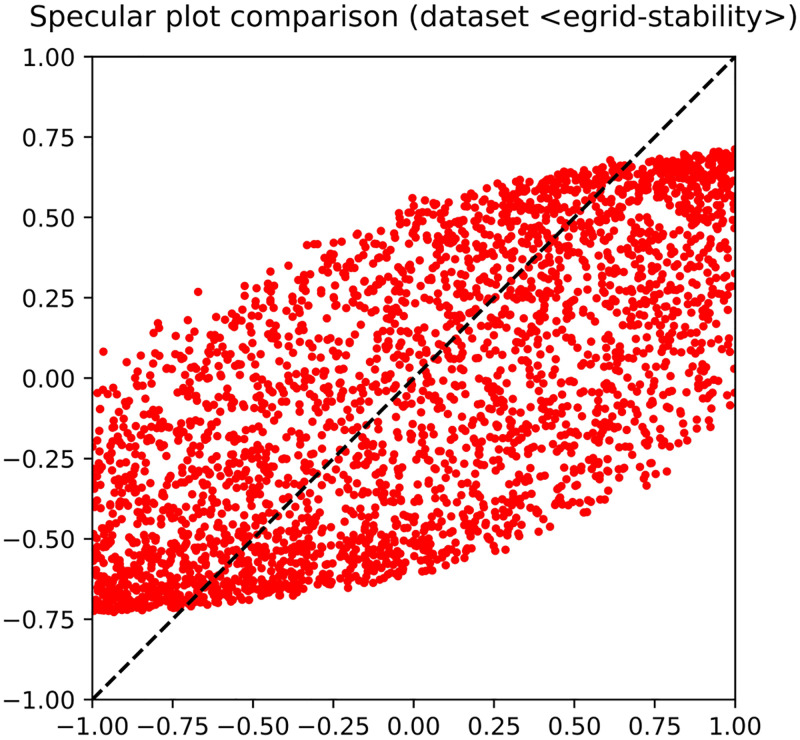
Observed-vs-predicted diagram that illustrates the lack of generalization obtained by the MLP on the dataset *EGrid stability*. The figure makes clear that the trained MLP performs a bad approximation of the given function.

As an example, Fig 5 shows the hidden layers and the output layer of the *EGrid stability* dataset. The figure makes clear that a pattern of failure has occurred therein, as no neurons have been generated able to reach a position close to the upper corner. Notably, the inability to fit the target is also stressed by the fact that no improvements occur across hidden layers. The corresponding observed-vs-predicted diagram confirms the validity of the analysis performed by means of 〈*φ*, *δ*〉 diagrams. In particular, Fig 6 highlights that an MLP trained on the cited dataset is far from reaching a good performance. The figure also highlights that the result of training has a visible bias with respect to the wanted optimal behavior arranged around the 45-degrees straight line.

As a concluding remark, although not being the main focus of this article, experimental results point out that progressive training performs well on most datasets, characterizing itself as a good alternative to other classical regressors.

## 4 Discussion

Evaluating the *CM* for a regression problem requires a change of perspective. Beyond technicalities, in presence of binary classification problems, the whole unitary token is delivered to only one among *TN*, *FP*, *FN*, and *TP*, depending on the target value and on whether the target and the feature value are coincident or not. A true token sharing strategy occurs when features are allowed to be real-valued. In this case either *TN* and *FP* or *FN* and *TP* are affected by the sharing, according to the sign of the target for the example at hand. The quantity assigned to the positive or negative side depends on the actual distance between the value *v* of the feature under analysis and the target value *t* (the closer, the better). For instance, with a target value *t* = −1 and a feature value *v* = −0.5 most of the unitary token would be assigned to *TN* and the remainder to *FP*. The generalization of token sharing to regression problems takes place by scaling the target in [−1, +1]. Hence, depending on its distance from −1 and +1, the actual target can be thought of as being *concurrently* negative and positive at the same time. In this case the token is first broken down in two parts, say *R*_⊖_ and *R*_⊕_, one to be delivered to the negative side (i.e., *TN* and *FP*) and the other to the positive side (i.e., *FN* and *TP*). Then *R*_⊖_ and *R*_⊕_ undergo a further break down, depending on the distance between the target and the feature value. Note that, according to this view, a target value *t* = 0 is in fact half negative and half positive, being equidistant from −1 and from +1. It is also worth pointing out that the given formulas are valid even when *t* = *v* = 0. In fact, in this case the equations reported in Table 4 would assign, as expected, half token to *TN* and half token to *TP*.

Focusing on the formation of characteristic patterns inside MLPs upon training, it is worth noting that contravariant neurons are barely found on regression problems. The motivation lies in the inherent difference between classification and regression tasks. In fact, both covariant and contravariant neurons may hold at the hidden layers of an MLP trained on a classification problem, for their ability of affecting the generalization process. When shifting to regression problems, contravariant patterns tend to be ruled out by the training algorithm, whose goal is to approximate as much as possible the target signal. As a consequence, most of the neurons that populate the hidden layers (typically all) are drifted towards the covariant side.

Notably, the ability of inspecting the hidden layers of an MLP with 〈*φ*, *δ*〉 measures and diagrams can also be helpful in the task of adapting its architecture to the given problem. Despite the growth of deep learning techniques, the research subfield of ANN architecture optimization is still very important, due to its capability to increase the performance of an ANN on the problem at hand. Without the claim of being exhaustive, some relevant proposals are briefly recalled hereinafter, also pointing to the fact that the history of ANN architecture and weight optimization is strongly interlaced with classical search heuristics. A historical approach based on Fogel’s evolutionary programming has been devised by Yao and Liu, 1997 [14]. Another exemplary case of this closeness can be found in the work of Zanchettin and Ludermir [15], who experimented a mix of simulated annealing, tabu search and genetic algorithms. This work is ideally related to the proposal of Ludermir and de Oliveira [16], in which particle swarm optimization was experimented. More recently, Ramchoun et al. [17] describe an approach based on genetic algorithms and mainly focused on connection deletion, as a way of increasing the ANN performance. Not least of all, the work of Fekri-Ershad and Ramakrishnan [18], still relating to genetic algorithms, use an innovative chromosome representation and cross-over process, devised to optimize the number of hidden layers and hidden nodes.

Along with this perspective, the proposed performance measure can be used for both classification and regression problems. In fact, in either case, some hidden layer neurons may reveal themselves as uneffective or even harmful to the intended purpose, depending on the values of *φ* and *δ*. Let us recall that the *φ*-axis (with equation *δ* = 0) is the locus of points with minimum mutual information between the neuron at hand and the target. This property holds despite the fact that the value of *φ* may carry different semantics. In particular, *φ* ≈ −1 and *φ* ≈ +1 mean that the neuron is emitting a fixed value (i.e., close to −1 or +1, respectively), regardless of the given inputs. Moreover, *φ* ≈ 0 (still in proximity of the *φ* axis) means that the neuron is emitting a random value. Summarizing, a 〈*φ*, *δ*〉 analysis can easily highlight when a neuron is not relevant (*φ* ≈ −1 or +1) or even harmful (*φ* ≈ 0 and |*δ*| ≈ 0) for the problem at hand. In these cases, the neuron can be removed in agreement with the selected optimization strategy.

## 5 Conclusions

This article has shown the feasibility of adopting 〈*φ*, *δ*〉 diagrams in the task of assessing the effectiveness of multilayer perceptrons trained on regression problems. To this end, a notable change in the policy used to evaluate the confusion matrix is made, with the goal of reporting information about regression performance therein. Moreover, it has been shown that the proposed policy maintains backwards compatibility with the previous definitions. The occurrence of success and failure patterns, together with the effectiveness of progressive training, has also been experimentally confirmed. As for future work, failure patterns will be further investigated –still on regression problems– with the aim of better illustrating the process that generates a failure during MLP training. Moreover, the generalized token sharing policy will be experimented on artificial datasets, to verify its effectiveness also under specific conditions devised to highlight pros and cons of the proposed approach. An attempt to integrate 〈*φ*, *δ*〉 measures in an optimization strategy devised to perform neural network pruning on regression problems is also under study.
